# Supplementary material for: Giant nonvolatile manipulation of magnetoresistance in magnetic tunnel junctions by electric fields via magnetoelectric coupling
Source: Nat Commun. 2019 Jan 16;10:243. doi: 10.1038/s41467-018-08061-5 (PMC6335399; doi:10.1038/s41467-018-08061-5)
Supplement: Supplementary file 1 — Supplementary information [file 41467_2018_8061_MOESM1_ESM.pdf]

Supplementary Information

Giant nonvolatile manipulation of magnetoresistance in magnetic tunnel junctions by electric fields via magnetoelectric coupling

Chen et al.

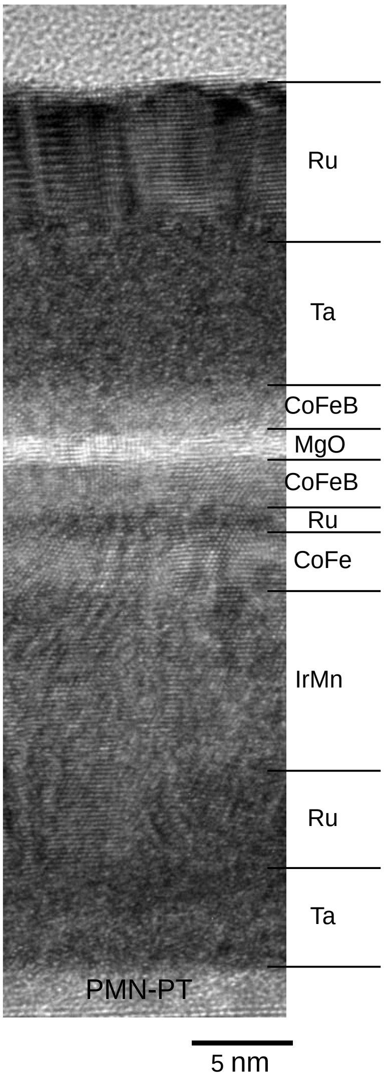

Supplementary Figure 1 | Cross-sectional HRTEM image for MTJ on PMN-PT. A cross-sectional HRTEM image of a typical MTJ devices. Each layer is readily distinguishable, suggesting that the MTJs have the designed structure. The scale bar is 5 nm.

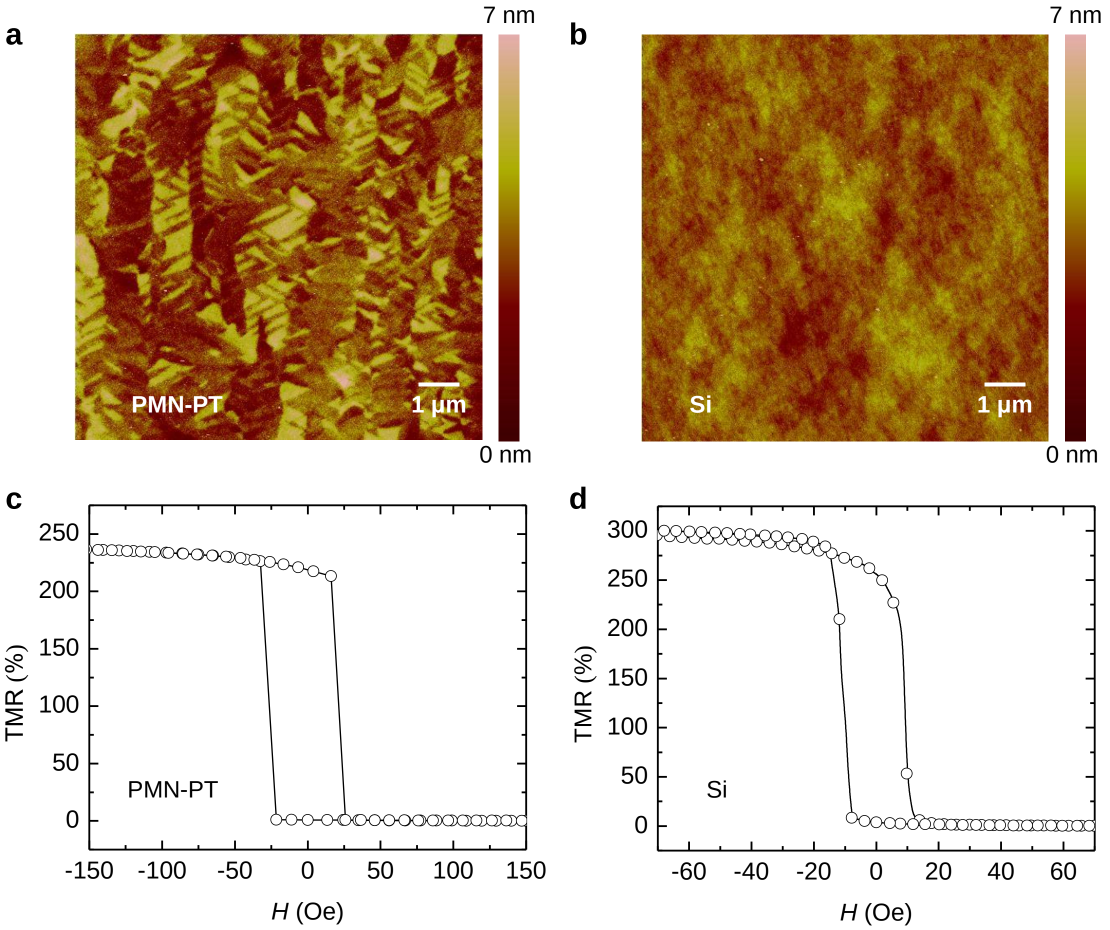

Supplementary Figure 2 | Comparisons of TMR curves for MTJs on PMN-PT (011) and Si wafer. a,b, Morphology of PMN-PT and Si taken by atomic force microscopy, respectively. It is obvious that surface roughness of PMN-PT is larger than that of silicon wafer. The scale bar is 1 μm. **c,d,** Typical TMR curves of MTJs on PMN-PT and Si, whose TMR ratios are nearly 240% and 300%, respectively. The TMR ratio is defined as $\left(R_{\uparrow\downarrow}-R_{\uparrow\uparrow}\right)/R_{\uparrow\uparrow}$, where $R_{\uparrow\uparrow}$ and $R_{\uparrow\downarrow}$ are the tunnel resistance when the magnetizations of the two electrodes are aligned in parallel and antiparallel, respectively. The small difference in TMR ratios of these two samples suggests that the surface roughness of PMN-PT does not affect much the quality of MTJs prepared on top.

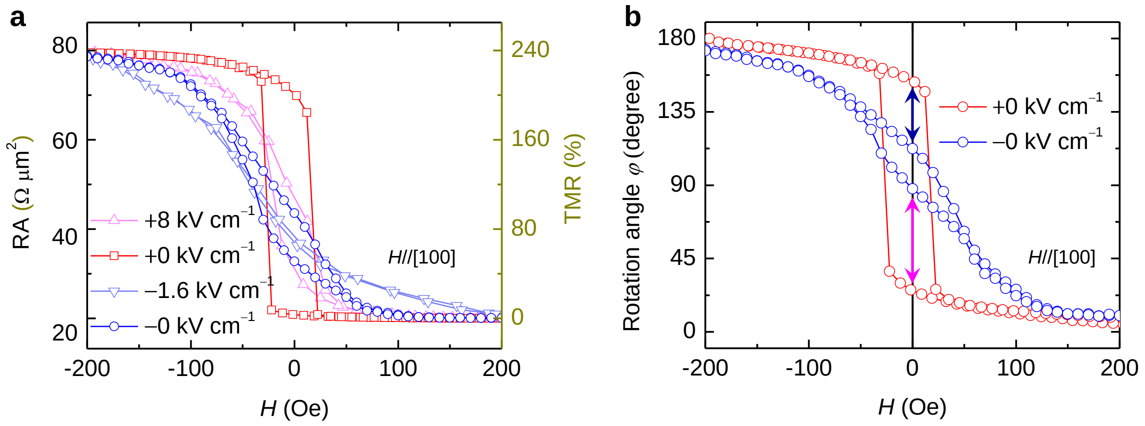

Supplementary Figure 3 | The striking effect of electric field in MTJs. a, Representative magnetoresistance curves measured in sequence at E = +8 kV cm^{-1}, +0 kV cm^{-1}, -1.6 kV cm^{-1} and -0 kV cm^{-1}. Obviously, the electric fields have a big effect on the magnetoresistance curves suggesting electrical control of magnetoresistance in MTJs. The resistance-area product RA plotted here is the tunnel resistance for a circular shape with a 10 μm diameter. **b,** The rotation angle of the free layer versus magnetic field for E = ±0 kV cm^{-1}. At H = 0 Oe, electric fields can rotate the magnetization of the free layer between parallel (antiparallel) and noncollinear as illustrated by the pink (blue) double-headed arrows.

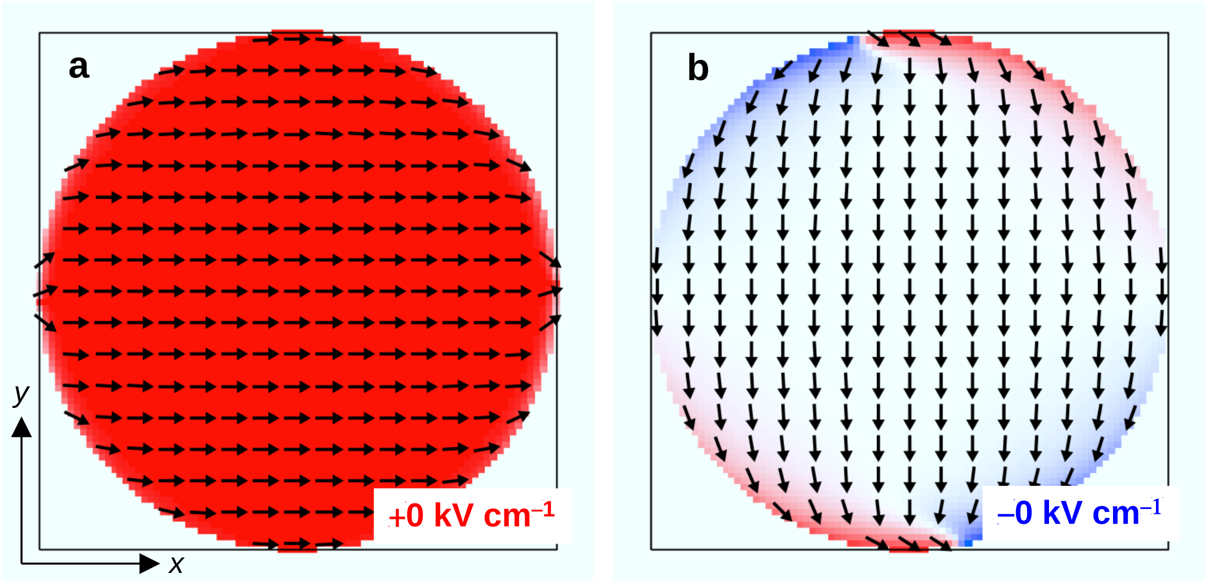

Supplementary Figure 4 | Micromagnetic simulation of ferromagnetic domain structure of the free layer at **a,** E = +0 kV cm^{-1} and **b,** E = –0 kV cm^{-1}.

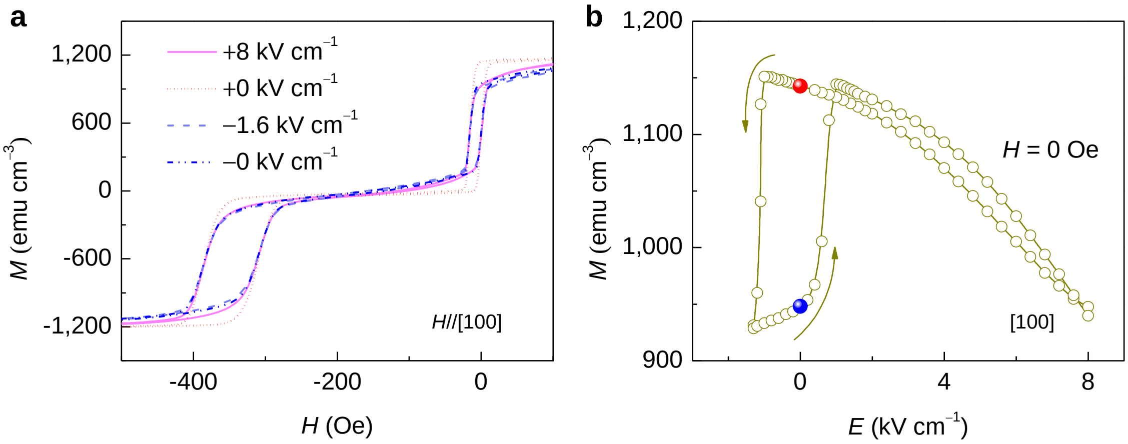

Supplementary Figure 5 | Magnetic characterizations of MTJ/PMN-PT under in situ electric fields. a, M-H loops of MTJ multilayers under E = +8 kV cm^{-1}, +0 kV cm^{-1}, -1.6 kV cm^{-1} and -0 kV cm^{-1}, respectively, which are measured along the [100] direction, i.e., the pinning direction. **b,** Electric-field dependence of magnetization at H = 0 Oe for Case II (inset of Fig. 2a in the main text) with a nonvolatile loop-like curve. There are two magnetization states at zero electric field.

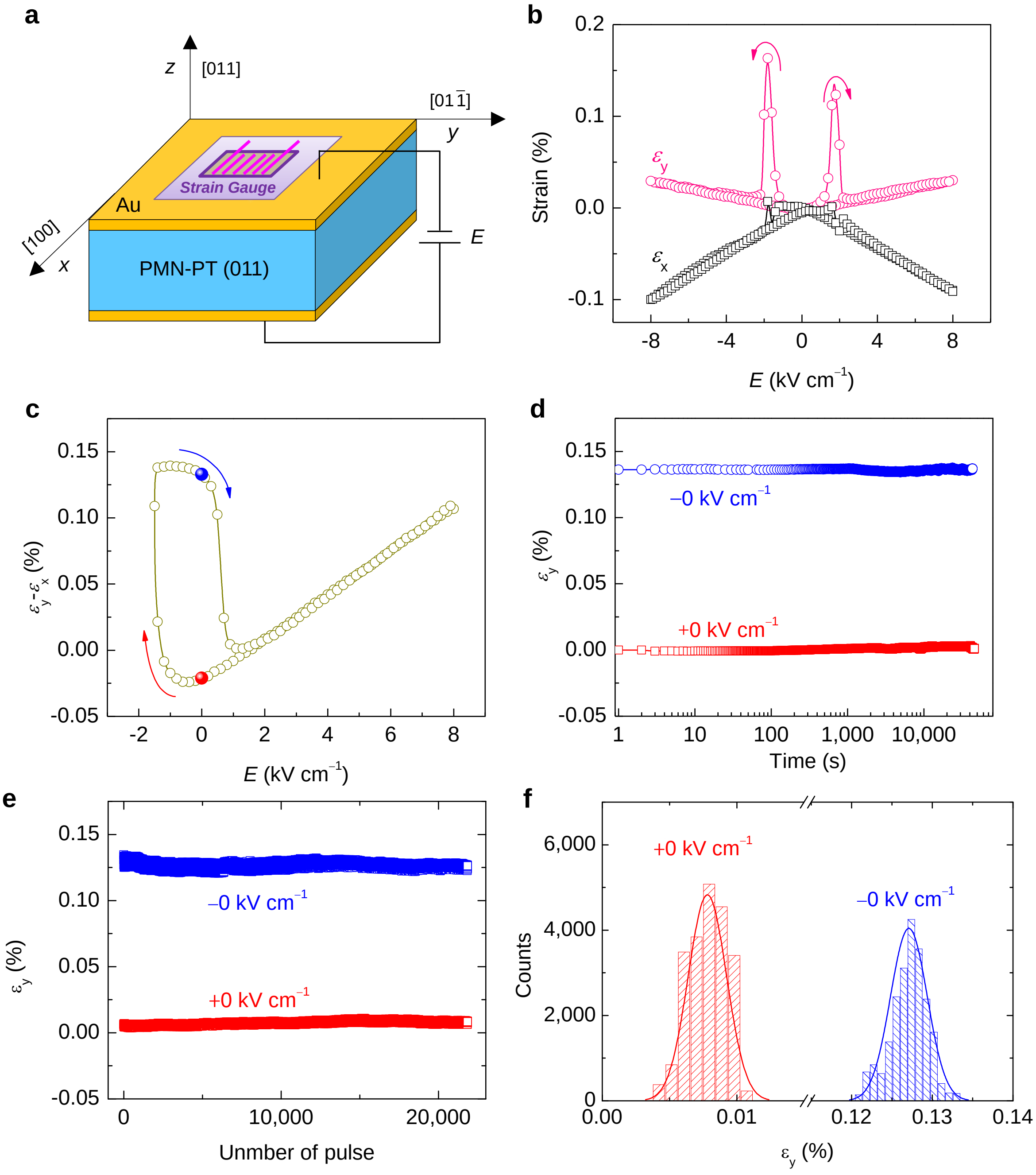

Supplementary Figure 6 | Strain property characterizations of the PMN-PT (011). a, Schematic of the sample and the experimental configuration for strain measurements using a strain gauge[1]. Sample edges *x*, *y* and *z* denote the pseudocubic [100], [01$\bar{1}$], and [011] crystallographic directions of PMN-PT, respectively. The voltage is exerted across the PMN-PT. **b,** ε_x and ε_y versus electric field curves measured along the [100] and [01$\bar{1}$] crystallographic directions of PMN-PT, respectively. The electric field is symmetric with ±8

kV cm^{-1}. Both ε_x and ε_y show a butterfly-like behavior without remanent strain at E = 0 kV cm^{-1}, which indicate the strain is volatile when applying symmetric electric fields with ±8 kV cm^{-1}. **c,** Dependence of anisotropic strain $\varepsilon_y - \varepsilon_x$ on asymmetric electric-field from 8 kV cm^{-1} to -1.6 kV cm^{-1}. The arrows in **b** and **c** indicate the electric-field sweeping directions. There are two distinctive strain states at E = ±0 kV cm^{-1} after applying E = 8 kV cm^{-1} and –1.6 kV cm^{-1}, respectively, suggesting the anisotropic strain is nonvolatile. **d,** The time dependent ε_y strain states at E = ±0 kV cm^{-1} measured over 13 hours indicate that these states are very stable. **e,** The reversible and stable nonvolatile strains stitched by 8 kV cm^{-1} and –1.6 kV cm^{-1} electric-field pulses for more than 20000 cycles. **f,** The strain distribution at ±0 kV cm^{-1} in **e**. This reversible and stable nonvolatile stain suggests the well reproducibility of ferroelectric domain states shown in Fig. 3.

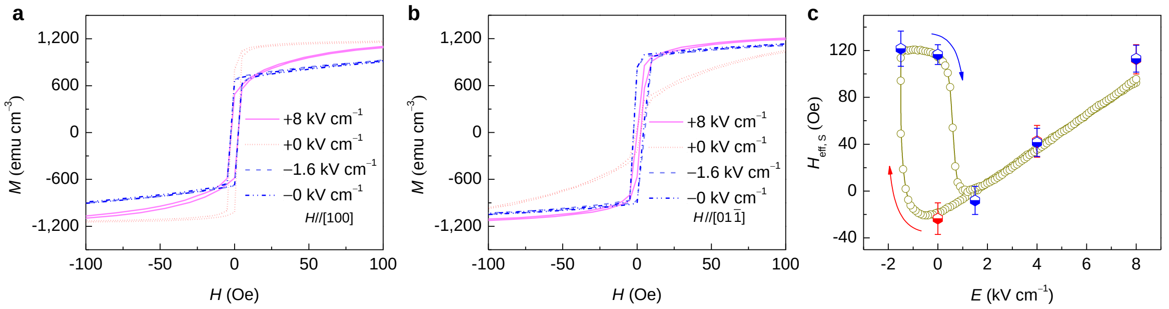

Supplementary Figure 7 | Electric-field modification of magnetic anisotropy for CoFeB film on PMN-PT (011). M-H loops of CoFeB film under different electric fields measured along **a,** the [100] direction and **b,** the $[01\bar{1}]$ direction. **c,** Electric-field dependence of strain-induced effective anisotropy field $H_{\mathrm{eff,\ S}}$. The points were extracted from **a** and **b**, and the line was derived from Supplementary Fig. 6c using $H_{\mathrm{eff,\ S}} = 3\lambda Y\left(\varepsilon_{\mathrm{y}} - \varepsilon_{\mathrm{x}}\right)/M_{\mathrm{S}}$.

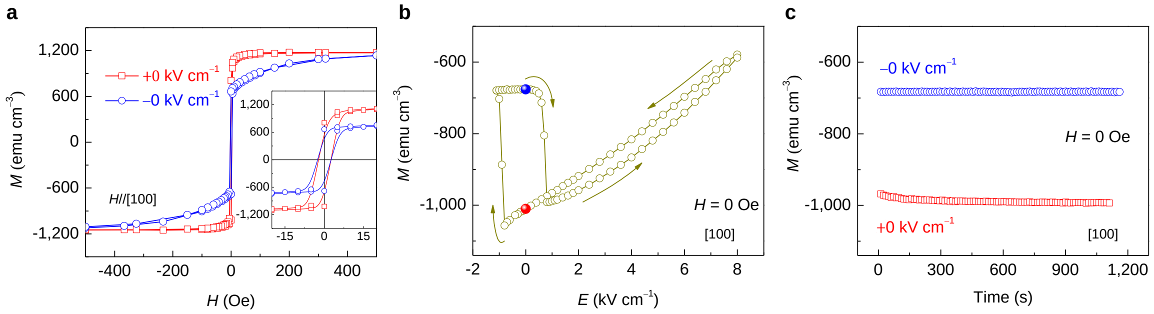

Supplementary Figure 8 | a, M-H loops at E = ±0 kV cm^{-1}. The inset shows the enlarged M-H loops around zero magnetic field. **b,** Magnetization versus electric field curve measured at H = 0 Oe with two distinctive magnetization states at E = 0 kV cm^{-1}. The measurements were performed at H = 0 Oe after initially applying –1000 Oe magnetic field. The arrows indicate the electric-field sweeping directions. **c,** The magnetizations at E = ±0 kV cm^{-1} after applying 8 kV cm^{-1} and –1.6 kV cm^{-1} remains almost the same. The bistable magnetization states at E = 0 kV cm^{-1} manifest reliable nonvolatile electrical control of magnetism. All data in the Figure were measured along the [100] direction.

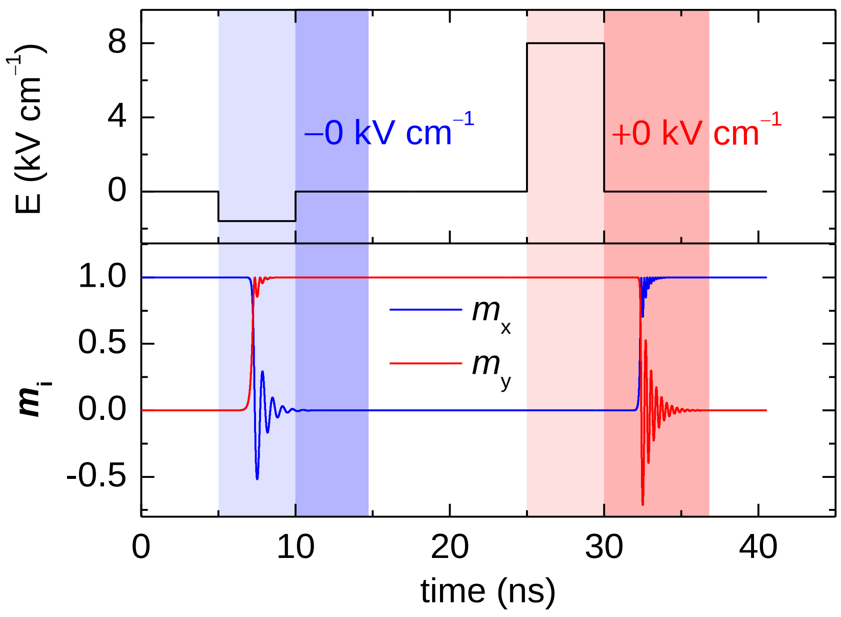

Supplementary Figure 9 | Magnetization rotation dynamic behavior driven by an electric-field pulse. The time scale indicates the real time of magnetization evolution solved from the Landau-Lifshitz-Gilbert (LLG) equation.

Supplementary Table 1 | The values of the rotation angles of the free layer at H = 0 Oe for E = ±0 kV cm^{-1} for Case I and Case II, respectively.

	+0 kV cm^{-1}	–0 kV cm^{-1}
Case I	155º	110º
Case II	23º	88º

Supplementary Note 1 | The values of the rotation angles of the free layer under electric fields.

According to Julliere's model[2], the dependence of resistance R of a MTJ unit on the rotation angle φ can be written as[3]:

$$R(\varphi)=\frac{R_c}{1+S_1S_2\cos\varphi} \tag{1}$$

where R_c is a coefficient, and S_1 and S_2 are the tunneling spin polarizations at Fermi level E_F of the two magnetic layers. For magnetization configurations of parallel and antiparallel, the corresponding rotation angles φ are 0° and 180°, respectively. So the resistances $R_{\uparrow\uparrow}$ and $R_{\uparrow\downarrow}$ can be expressed as follows:

$$\begin{cases} R_{\uparrow\uparrow}=\dfrac{R_c}{1+S_1S_2} \\ R_{\uparrow\downarrow}=\dfrac{R_c}{1-S_1S_2} \end{cases} \tag{2}$$

The values of $R_{\uparrow\uparrow}$ and $R_{\uparrow\downarrow}$ can be gotten from Supplementary Fig. 3a, so R_c and S_1S_2 also can be calculated using Supplementary Equation (2). Then using Supplementary Equation (1), the rotation angle can be obtained at certain $R(\varphi)$ and it can be written as:

$$\varphi=\arccos\left(\frac{R_c/R(\varphi)-1}{S_1S_2}\right) \tag{3}$$

Using Supplementary Equation (3), the rotation angles of the free layer around zero magnetic field for E = ±0 kV cm^{-1} in Supplementary Fig. 3b were deduced from corresponding resistances in Supplementary Fig. 3a. Supplementary Table 1 shows the values of the rotation angles of the free layer at H = 0 Oe for E = ±0 kV cm^{-1} for Case I and Case II, respectively.

Moreover, we performed micromagnetic simulations using the object-oriented

micromagnetic framework (OOMMF) software to study the domain evolution of the free layer under electric fields. Similar to the device structure, a circular shaped CoFeB disk of 10 μm in diameter was built and discretized in the computational cells of 10 nm×10 nm. The saturation magnetization, M_S, is 1200 emu cm^{-3}; and the uniform exchange constant, A, is 2.8 × 10^{-11} J m^{-1} (ref.[4]). To simulate the domain structures of the free layer of the MTJ under electric fields, a single domain along the [100] direction of PMN-PT (the pinning direction of the MTJs) was set as the initial state. Supplementary Fig. 4 shows the domain structures at E = ±0 kV cm^{-1}. For E = +0 kV cm^{-1}, the magnetization mainly points to the x direction while the magnetization rotates to the y direction for E = –0 kV cm^{-1}. But the magnetization around the edge is not rotated by 90°. This may be one reason that the rotation angles of the free layer at H = 0 Oe for E = ±0 kV cm^{-1} are not actually 90° as shown in Supplementary Table 1. Additionally, the TMR curve at E = +0 kV cm^{-1} (Fig. 1c) is not square and sharp suggesting the existence of multidomain in the micrometer-sized circular-shaped MTJ. This also can lead to the rotation angle of the free layer smaller than 90°.

Supplementary Note 2 | Electric-field modification of magnetic anisotropy for CoFeB film on PMN-PT.

To study the effect of this nonvolatile strain on magnetic anisotropy of the CoFeB film, single CoFeB film was sputtered on PMN-PT (011). The structure of single layer PMN-PT/CoFeB sample is PMN-PT/Ta(5)/Cu(10)/Ta(5)/CoFeB(4)/Ta(5) (numbers are nominal thicknesses in nanometres) with a size of 5×3×0.5 mm^3. The piezostrain of the FE substrate can transfer to the FM film modifying its magnetic anisotropy via the converse magnetostriction effect[5,6]. Routinely, as shown in Supplementary Figs. 6a and 6b, the M-H

loops under different electric fields with a remarkable change were measured along the [100] direction and the $[01\bar{1}]$ direction, respectively. As shown in Supplementary Fig. 7a, the magnetization process of the sample along the [100] direction at E = –0 kV cm^{-1} becomes harder and the remnant magnetization reduces than that at E = +0 kV cm^{-1}. However, the situation along the $[01\bar{1}]$ direction (Supplementary Fig. 7b) is just the converse, with an increment of the M-H squareness under E = –0 kV cm^{-1}. The different behaviors of M-H loops in Supplementary Figs. 7a and 7b indicate that the magnetic easy axis is along the [100] direction at E = +0 kV cm^{-1} while along the $[01\bar{1}]$ direction at E = –0 kV cm^{-1}.

To gain further insight into the electric-field-tuned magnetic anisotropy, the effective anisotropy fields $H_{\rm eff}$ at various electric fields were extracted from the M-H loops[7,8] in Supplementary Figs. 7a and 7b. It should be pointed out that the experimental piezostrain-induced effective anisotropy field $H_{\rm eff,\,S}$ (hexagon in Supplementary Fig. 7c) has subtracted a uniaxial magnetic anisotropy about 90 Oe because of the initial randomness of FE domains in PMN-PT[9]. The strain-induced effective anisotropy field can be written as[8-11] $H_{\rm eff,\ S} = 3\lambda Y\left(\varepsilon_{\rm y} - \varepsilon_{\rm x}\right)/M_{\rm S}$, where λ, Y and $M_{\rm S}$ are the magnetostriction coefficient, Young's modulus and saturation magnetization of CoFeB, respectively. Supplementary Fig. 7c plots $H_{\rm eff,\ S}$ as a function of electric field derived from the anisotropic strain $\varepsilon_{\rm y} - \varepsilon_{\rm x}$ (Supplementary Fig. 6c) using $M_{\rm S}$ = 1150 emu cm^{-3} deduced from the M-H loops in Supplementary Fig. 7a and the typical values for CoFeB thin film[9,10] with Y = 160 GPa and λ = 3×10^{-5}. The excellent agreements between the experimental and theoretical results confirm the efficient transfer of strain from PMN-PT to CoFeB thin film, which changed its magnetic anisotropy.

Owing to this giant electric-field-tuned magnetic anisotropy, the electric field can significantly tune the magnetization state of the FM thin film. Supplementary Fig. 8a shows the M-H loops measured along the [100] direction at E = ±0 kV cm^{-1}, respectively, which have a remarkable change. Supplementary Fig. 8b presents magnetization versus electric field curve measured at H = 0 Oe. Importantly, this nonvolatile electric-field-controlled magnetism has two distinct magnetization states after removing the asymmetric electric fields at E = 0 kV cm^{-1} and these two magnetization states are very stable as shown in Supplementary Fig. 8c. This nonvolatile electrical control of magnetism in CoFeB is certainly the cornerstone of reversible and reliable nonvolatile electrical manipulation of magnetoresistance of the MTJs in Fig. 1f.

These results can be understood by the anisotropic strain of the PMN-PT (011) in Supplementary Fig. 6c as follows. In Supplementary Fig. 6c, the anisotropic strain at E = +0 kV cm^{-1} after applying +8 kV cm^{-1} is negative, which is compressive strain along y axis, so the magnetic easy axis is along the [100] direction considering the positive magnetostriction coefficient of CoFeB film[9,10]. In contrast, the anisotropic strain at E = –0 kV cm^{-1} after applying −1.6 kV cm^{-1} is positive, which is tensile strain along y axis, so the strain-induced magnetic anisotropy is along the [$01\bar{1}$] direction leading to the rotation of magnetic easy axis. Thus through strain-mediated magnetoelectric coupling, the magnetic easy axis of CoFeB film can be reversibly switched by applying asymmetric electric fields, which can modify the magnetization.

Supplementary Note 3 | Estimation of the width of electric-field pulse.

The width of electric-field pulse depends on the time that is required to switch the

magnetization of the free layer. This time mainly includes three parts: the average magnetization switching time t_{FM} of the free layer in MTJ, the ferroelectric polarization switching time t_{FE} to produce nonvolatile strain, and the time t_{strain} for strain transfer from the ferroelectric layer to the free layer.

1), To estimate t_{FM}, we performed a macro-spin model simulation based on the conventional Landau-Lifshitz-Gilbert (LLG) equation to investigate the dynamics of the magnetization. The LLG is expressed as follows[8,12,13]:

$$\frac{\mathrm{d}\mathbf{m}}{\mathrm{d}t} = -\gamma \mathbf{m} \times \mathbf{H}_{\mathrm{eff}} + \alpha \mathbf{m} \times \frac{\mathrm{d}\mathbf{m}}{\mathrm{d}t} \quad (4)$$

Where γ is the gyromagnetic ratio, α is the damping constant and **m** is a cosine vector of magnetization $\mathbf{m} = (m_x, m_y, m_z)$. The typical values[8,13,14] are $\gamma = 1.7 \times 10^7$ Hz Oe^{-1} and $\alpha = 0.01$. $\mathbf{H}_{\mathrm{eff}}$ is the effective field, which is obtained from the magnetic anisotropy energies, and can be expressed as follows:

$$\mathbf{H}_{\mathrm{eff}} = -\nabla_m \left[\frac{1}{2} H_0 m_x^2 + \frac{1}{2} H_{\mathrm{eff,\,S}} m_y^2 + \frac{1}{2} H_{\perp} m_z^2 \right] \quad (5)$$

Here, H_0 (about 90 Oe) is the magnetic anisotropy field resulting from the initial randomness of ferroelectric domains in PMN-PT (ref.[9]). $H_{\mathrm{eff,S}}$ is the strain-induced effective anisotropy field, the out-of-plane effective anisotropy field $H_{\perp}$ is about 1 T as shown by our previous electron spin resonance results[15]. The dipole field from the pinned layer is neglected assuming that this dipole field can be cancelled out by synthetic ferromagnetic reference layer[16].

Supplementary Fig. 9 presents the simulation results of magnetization rotation under electric fields. The −0 kV cm^{-1} state is obtained by applying an electric field of −1.6 kV cm^{-1}. It includes two steps, i.e., applying −1.6 kV cm^{-1} and removing it. It can be seen from

Supplementary Fig. 9 that the magnetization takes about 5 ns to rotate to the *y* direction and stabilizes at the *y* direction after removing −1.6 kV cm^{-1}. The total time is less than 10 ns. Similarly, the +0 kV cm^{-1} state is obtained by applying and then removing an electric field of 8 kV cm^{-1}. From –0 kV cm^{-1} to 8 kV cm^{-1}, because the magnetic easy axes of both of them are along the *y* direction and the magnetization almost does not change, the time for magnetization rotation is neglected as shown in Supplementary Fig. 9 and the width of voltage pulse is determined by the time for ferroelectric domain switching from the in-plane to the out-of-plane. When removing electric field to +0 kV cm^{-1}, the magnetization takes less than 10 ns to rotate back to the *x* direction. Thus the time for magnetization rotation is less than 10 ns for both –0 kV cm^{-1} state and +0 kV cm^{-1}.

2), The t_{FE} is usually below 10 ns and the polarization normally switches much faster than the magnetization[17,18].

3), The t_{strain} can be estimated by[19]

$$t_{\text{strain}} = \frac{d}{v} \tag{6}$$

Where *d* is the distance between ferroelectric and the free layer, and *v* is the velocity of sound in the buffer layer. It is well known that the speed of sound varies from substance to substance, but generally it travels very fast in solids. We assume v = 3000 m s^{-1} (ref.[17]). So $t_{strain} \approx$ 0.009 ns for d = 27 nm.

In summary, the width of voltage pulse is less than 20 ns for our devices. In addition, the theoretical work on multiferroics heterostructures using ferroelectric films reported that the strain-induced switching of multiferroic nanomagnets can be less than 10 ns (refs.[17,20]) and even less than 1 ns (ref.[21]).

Supplementary Note 4 | Calculation of the power consumption for electrical manipulation of MR in MTJs.

In our devices, the power consumption per unit area can be estimated by CV^2/A (ref.[20,21,22]), where C, V and A denote the capacitance of the piezoelectric layer, the applied voltage and the area of the device, respectively. The capacitance can be written as $C = \varepsilon_r\varepsilon_0 A/d$ (ref.[22]), assuming a parallel plate capacitor (A is the area of the electrode, d is the thickness of the piezoelectric layer, ε_r is the relative dielectric constant of the piezoelectric and ε_0 is the vacuum dielectric constant). Thus the power consumption per unit area can be expressed as $\varepsilon_r\varepsilon_0 V^2/d$. In our devices, the operation electric fields for ±0 kV cm^{-1} states in Fig. 1 are 8 kV cm^{-1} and –1.6 kV cm^{-1}, so the operation voltages are 400 V and –80 V, respectively, considering the 500 μm thickness of PMN-PT substrate. The relative dielectric constant ε_r of PMN-PT is about 3000 (ref.[23]). So the power consumptions per unit area are about 0.85 mJ cm^{-2} and 0.034 mJ cm^{-2} for ±0 kV cm^{-1} states, respectively.

The state-of-the-art spin-transfer-torque magnetic tunnel junctions require about a 0.7 V voltage pulse of 500 ps (ref.[24]) or 120 ps (ref.[25]) in duration through a 60-70 nm×180 nm device, producing an energy dissipation per unit area of 3-4 mJ cm^{-2}. So the power consumption per unit area in our devices is remarkably smaller than that of the state-of-the-art spin-transfer-torque devices. Additionally, there are also some theoretical work reporting that ultralow power dissipation can be achieved in multiferroics heterostructures using ferroelectric films[17,20,26]. For example, energy dissipation per unit area can be reduced to 4 μJ cm^{-2} with high-speed operation below 10 ns (ref.[17]). High quality PMN-PT epitaxial thin films on Si wafers with giant piezoelectricity have been reported[27], which makes our work more attractive for future applications. Our present work shows that integrating spintronics and

multiferroics is a reachable way to achieve nonvolatile electrical manipulation of MTJs solely by electric fields without the assistance of a magnetic field and also the potential to reduce energy consumption.

Supplementary References

1. Yang, L. *et al.* Bipolar loop-like non-volatile strain in the (001)-oriented $Pb(Mg_{1/3}Nb_{2/3})O_3$-$PbTiO_3$ single crystals. *Sci. Rep.* **4,** 4591 (2014).
2. Julliere, M. Tunneling between ferromagnetic films. *Phys. Lett. A* **54,** 225-226 (1975).
3. Hu, J., Li, Z., Wang, J. & Nan, C. W. Electric-field control of strain-mediated magnetoelectric random access memory. *J. Appl. Phys.* **107,** 093912 (2010).
4. Li, P. *et al.* Spatially resolved ferroelectric domain-switching-controlled magnetism in $Co_{40}Fe_{40}B_{20}/Pb(Mg_{1/3}Nb_{2/3})_{0.7}Ti_{0.3}O_3$ multiferroic heterostructure. *ACS Appl. Mater. Interfaces* **9,** 2642-2649 (2017).
5. Ma, J., Hu, J., Li, Z. & Nan, C. Recent progress in multiferroic magnetoelectric composites: from bulk to thin films. *Adv. Mater.* **23,** 1062-1087 (2011).
6. Hu, J., Chen, L. & Nan, C. Multiferroic heterostructures integrating ferroelectric and magnetic materials. *Adv. Mater.* **28,** 15-39 (2016).
7. Wang, K. Y. *et al.* Spin reorientation transition in single-domain (Ga,Mn)As. *Phys. Rev. Lett.* **95,** 217204 (2005).
8. Li, P. *et al.* Electric field manipulation of magnetization rotation and tunneling magnetoresistance of magnetic tunnel junctions at room temperature. *Adv. Mater.* **26,** 4320-4325 (2014).
9. Zhang, S. *et al.* Giant electrical modulation of magnetization in $Co_{40}Fe_{40}B_{20}/Pb(Mg_{1/3}Nb_{2/3})_{0.7}Ti_{0.3}O_3(011)$ heterostructure. *Sci. Rep.* **4,** 3727 (2014).

10. Chen, A. *et al.* Angular dependence of exchange bias and magnetization reversal controlled by electric-field-induced competing anisotropies. *Adv. Mater.* **28,** 363-369 (2016).

11. Chen, A. T. & Zhao, Y. G. Electrical manipulation of magnetism through strain-mediated magnetoelectric coupling in multiferroic heterostructures. *APL Mater.* **4,** 032303 (2016).

12. Shiota, Y. *et al.* Induction of coherent magnetization switching in a few atomic layers of FeCo using voltage pulses. *Nat. Mater.* **11,** 39-43 (2012).

13. Maruyama, T. *et al.* Large voltage-induced magnetic anisotropy change in a few atomic layers of iron. *Nat. Nanotechnol.* **4,** 158-161 (2009).

14. Ikeda, S. *et al.* A perpendicular-anisotropy CoFeB-MgO magnetic tunnel junction. *Nat. Mater.* **9,** 721-724 (2010).

15. Zhang, S. *et al.* Electric-field control of nonvolatile magnetization in $Co_{40}Fe_{40}B_{20}/Pb(Mg_{1/3}Nb_{2/3})_{0.7}Ti_{0.3}O_3$ structure at room temperature. *Phys. Rev. Lett.* **108,** 137203 (2012).

16. Sato, H. *et al.* Co/Pt multilayer based reference layers in magnetic tunnel junctions for nonvolatile spintronics VLSIs. *Jpn. J. Appl. Phys.* **53,** 04EM02 (2014).

17. Hu, J., Li, Z., Chen, L. & Nan, C. High-density magnetoresistive random access memory operating at ultralow voltage at room temperature. *Nat. Commun.* **2,** 553 (2011).

18. Kohlstedt, H. *et al.* Current status and challenges of ferroelectric memory devices. *Microelectron. Eng.* **80,** 296-304 (2005).

19. Schröder, K. Stress operated random access, high-speed magnetic memory. *J. Appl. Phys.* **53,** 2759-2761 (1982).

20. Roy, K., Bandyopadhyay, S. & Atulasimha, J. Hybrid spintronics and straintronics: A

magnetic technology for ultra low energy computing and signal processing. *Appl. Phys. Lett.* **99,** 063108 (2011).

21. Roy, K., Bandyopadhyay, S. & Atulasimha, J. Energy dissipation and switching delay in stress-induced switching of multiferroic nanomagnets in the presence of thermal fluctuations. *J. Appl. Phys.* **112,** 023914 (2012).

22. Biswas, A. K., Ahmad, H., Atulasimha, J. & Bandyopadhyay, S. Experimental demonstration of complete 180° reversal of magnetization in isolated Co nanomagnets on a PMN-PT substrate with voltage generated strain. *Nano Lett.* **17,** 3478-3484 (2017).

23 Wu, T. *et al.* Electrical tuning of metastable dielectric constant of ferroelectric single crystals for low-power electronics. *Appl. Phys. Lett.* **99,** 182903 (2011).

24 Liu, H. *et al.* Ultrafast switching in magnetic tunnel junction based orthogonal spin transfer devices. *Appl. Phys. Lett.* **97,** 242510 (2010).

25. Rowlands, G. E. *et al.* Deep subnanosecond spin torque switching in magnetic tunnel junctions with combined in-plane and perpendicular polarizers. *Appl. Phys. Lett.* **98,** 102509 (2011).

26. Roy, K. Ultra-low-energy non-volatile straintronic computing using single multiferroic composites. *Appl. Phys. Lett.* **103,** 173110 (2013).

27. Baek, S. H. et al. Giant piezoelectricity on Si for hyperactive MEMS. Science **334,** 958-961 (2011).
